# Physiological and Pharmacological Roles of FGF21 in Cardiovascular Diseases

**DOI:** 10.1155/2016/1540267

**Published:** 2016-05-09

**Authors:** Peng Cheng, Fangfang Zhang, Lechu Yu, Xiufei Lin, Luqing He, Xiaokun Li, Xuemian Lu, Xiaoqing Yan, Yi Tan, Chi Zhang

**Affiliations:** ^1^The Chinese-American Research Institute for Diabetic Complications, Wenzhou Medical University, Wenzhou 325035, China; ^2^Ruian Center of the Chinese-American Research Institute for Diabetic Complications, The Third Affiliated Hospital, Wenzhou Medical University, Wenzhou 325200, China; ^3^School of Pharmaceutical Sciences, Wenzhou Medical University, Wenzhou 325035, China; ^4^Kosair Children Hospital Research Institute, Department of Pediatrics, University of Louisville School of Medicine, Louisville, KY 40202, USA

## Abstract

Cardiovascular disease (CVD) is one of the most severe diseases in clinics. Fibroblast growth factor 21 (FGF21) is regarded as an important metabolic regulator playing a therapeutic role in diabetes and its complications. The heart is a key target as well as a source of FGF21 which is involved in heart development and also induces beneficial effects in CVDs. Our review is to clarify the roles of FGF21 in CVDs. Strong evidence showed that the development of CVDs including atherosclerosis, coronary heart disease, myocardial ischemia, cardiac hypertrophy, and diabetic cardiomyopathy is associated with serum FGF21 levels increase which was regarded as a compensatory response to induced cardiac protection. Furthermore, administration of FGF21 suppressed the above CVDs. Mechanistic studies revealed that FGF21 induced cardiac protection likely by preventing cardiac lipotoxicity and the associated oxidative stress, inflammation, and apoptosis. Normally, FGF21 induced therapeutic effects against CVDs via activation of the above kinases-mediated pathways by directly binding to the FGF receptors of the heart in the presence of *β*-klotho. However, recently, growing evidence showed that FGF21 induced beneficial effects on peripheral organs through an indirect way mediated by adiponectin. Therefore whether adiponectin is also involved in FGF21-induced cardiac protection still needs further investigation.

## 1. Introduction

Cardiovascular diseases (CVDs) are the leading cause of death worldwide composed of heart and blood vessel diseases. In the recent years, the incidence of CVDs has been increasing at a sharp rate globally. According to the World Health Report 2010, CVDs contributed to 17.5 million deaths and these numbers are estimated to increase to 23.3 million by 2030 [1, 2].

Fibroblast growth factor (FGF) is a cytokine superfamily with pleiotropic biological functions including regulating cell growth, differentiation, development, and metabolism [3–7]. Human FGFs contain 22 members which can be divided into 7 subfamilies based on phylogeny and sequence [8–10]. Due to the lack of a heparin binding domain, FGF19 subfamily members (FGF19, FGF21, and FGF23) function in an endocrine manner rather than an autocrine manner as other subfamily members of FGFs [9]. Among them, FGF21 is a polypeptide with 209/210 (human/rodent) amino acid residues that is primarily produced and secreted by the liver, adipose tissue, and thymus [11]. FGF21 expression is mainly regulated by peroxisome proliferator-activated receptor *α* (PPAR*α*) in the liver [12] and PPAR*γ* in adipocytes [13, 14]. FGF21 was firstly cloned in 2000 [11] and received global attention in recent years due to its outstanding ability on regulating carbohydrate and lipid metabolism including improving insulin sensitivity, lowering blood glucose, reducing hepatic/plasma triglycerides, inducing weight loss by increasing energy expenditure, and reducing fat mass [15–18]. Further studies indicated that FGF21 functions by binding to (FGFR)1c and (FGFR)2c in the presence of coreceptor *β*-klotho and activation of downstream signaling pathway [19, 20]. Although FGF21 and other members of FGFs share the same FGF receptors, the coreceptors are different (*β*-klotho for FGF21 and heparin for others) which determined that they have different bioactivity due to activation of various pathways [21, 22]. Unlike traditional insulin therapy in clinics, FGF21 did not cause hypoglycemia [16]. The possible explanation is that FGF21 induces physiological role in healthy condition and pharmacological role under unhealthy condition [23, 24]. Additionally, FGF21 does not lead to carcinogenic event due to lack of mitogenic function which makes it possible to be administrated* in vivo* in clinics [16]. Therefore, FGF21 may hold promise as a clinically therapeutic option due to the abovementioned characters and advantages.

In recent clinical and preclinical studies, CVDs have been closely associated with serum FGF21 which increased in the patients with atherosclerosis, coronary heart disease, myocardial ischemia, cardiac hypertrophy, and diabetic cardiomyopathy [25–27]. Therefore FGF21 has the potential to be considered as a biomarker for the above CVDs.

Whether the increased serum FGF21 level is the basis for CVD pathogenesis or is induced to protect the heart form CVDs is still under discussion. However, growing evidence indicated that administration of exogenous FGF21 induces preventive effects on most of the above CVDs, suggesting that FGF21 not only is a simple marker of cardiovascular risk but also induces a protective effect on the cardiovascular system contributing to a reduction in risk (Table 1). In clinics, serum FGF21 levels were increased in patients with obesity or type 2 diabetes which was associated with high risk of CVDs. The paradoxical phenomenon was supposed to be explained by a compensatory response to induce cardiac protection or resistance to FGF21 which impaired its bioactivity [28, 29]. In animal study, we found that at the early-stage of diabetes serum FGF21 level of mice was sharply increased compared with nondiabetic mice (C57BL/6J), while it was dramatically decreased at the late-stage of diabetes which further confirmed that early-stage increase of serum FGF21 was a compensatory response and induced beneficial effect on the heart; late-stage decrease may be the cause of diabetes-induced cardiac damage [30], since the above CVDs are always attributed to lipid metabolic disorder. Mechanistic studies indicated that FGF21-induced cardiac protection in CVDs is possibly attributed to the suppression of lipotoxicity since the above CVDs are always the consequences of lipotoxicity. This review tries to illuminate the underlying relationship between FGF21 and CVDs and the possible mechanisms.

## 2. FGF21 and Atherosclerosis and Coronary Heart Disease

Atherosclerosis is a chronic, inflammatory disorder characterized by the deposition of excess lipids in the arterial intima [31]. The accrued evidence indicated that lipid-lowering therapy limits the progression of atherosclerosis and reduces CAD events [32]. Since FGF21 plays an important role in the regulation of lipid metabolism, the effect of FGF21 in atherosclerosis is of interest. Clinical studies showed that increased circulating FGF21 levels were discovered in atherosclerotic patients or the individuals with high risk of developing atherosclerosis [33, 34]. Additionally, an* in vivo* study demonstrated that increased serum FGF21 was observed in aortas of apoE^−/−^ mice (C57BL/6J background) [35]. Strong evidence identified that administration of exogenous FGF21 significantly improved lipid metabolic disorders and reduced atherosclerotic plaque areas in these animals [36]. Moreover, Lin et al. also reported that FGF21 deficiency enhanced atherosclerotic deterioration and mortality in apoE^−/−^ mice (C57BL/6J background) [35], implying that increased serum FGF21 in patients with atherosclerosis described previously induces beneficial effect rather than the basic for atherosclerotic pathogenesis. Mechanistic study indicated that FGF21-induced prevention of atherosclerosis was associated with suppression of endoplasmic reticulum stress-mediated apoptosis in apoE^−/−^ mice (C57BL/6J background) [37]. Further mechanistic studies revealed that prevention of atherosclerosis by FGF21 was attributed to the fine-tuning of multiorgan cross talk among the liver, adipose tissue, and blood vessels, characterized by suppression of hepatic sterol regulatory element-binding protein-2 and induction of adiponectin in mice with atherosclerosis [35]. Although FGF21 functions in an endocrine manner, whether FGF21 can also induce a direct protection to the blood vessels remains unclear. For decades, lowering levels of low-density lipoprotein (LDL) cholesterol and increasing level of high-density lipoprotein (HDL) have formed the cornerstone of management of patients with atherosclerotic cardiovascular disease. Strong evidence demonstrated that FGF-21 dramatically improved the condition of atherosclerosis in Wistar rats by decreasing serum LDL levels and increasing serum HDL levels. Moreover, FGF-21-induced antioxidative function is also involved in its therapeutic effect in atherosclerotic Wistar rat characterized by increased levels of superoxide dismutase, reduced glutathione, and reduced malondialdehyde [38].

Along with the development of atherosclerosis, the artery's lining becomes hardened, stiffened, and swollen with all sorts of “gunge,” including fatty deposits and abnormal inflammatory cells, to form a plaque and then eventually deteriorate into coronary heart disease [39–41]. Strong evidence indicated that cardiac endothelial cell dysfunction may be an early initiating factor for atherosclerosis which facilitates the development of coronary heart disease [42]. Oxidized LDL (ox-LDL) is a proatherogenic lipoprotein that accumulates in the vascular wall and contributes to vascular dysfunction at the early-stage of atherosclerosis development [43–53]. Enhanced serum ox-LDL and antibodies against its epitopes are predictive for endothelial dysfunction and subsequent coronary heart disease [43]. Previous* in vitro* study indicated that both FGF21 mRNA and protein expressions were increased in response to ox-LDL treatment in cardiac endothelial cells and this was protective against apoptosis caused by ox-LDL [54]. Also, FGF21 has been reported to prevent high glucose induced cell damage and endothelial nitric oxide synthase dysfunction through an AMP-activated protein kinase- (AMPK-) dependent pathway in endothelial cells [55]. Therefore the relationship between FGF21 and coronary heart disease is of interest. Shen et al. reported that serum FGF21 level was positively associated with coronary heart disease in clinics [56, 57]. Our previous work confirmed that serum levels of FGF-21 are increased in patients with coronary heart disease independently associated with adverse lipid profiles [33]. In contrast, another study indicated that serum FGF21 has been associated with hypertriglyceridemia, hyperinsulinemia, and pericardial fat accumulation but not associated with coronary heart disease [58]. This paradox may be explained by decreased body mass index of healthy controls compared to patients with coronary heart diseases.

## 3. FGF21 and Myocardial Ischemia

Myocardial ischemia, a disorder causing cardiomyocytes injury and myocardial infarction and malfunction, activates adaptive responses enhancing myocardial tolerance to ischemia. Liu et al. indicated that, in response to myocardial ischemia in the C57BL/6J mouse, liver- and adipocytes-derived FGF21 was upregulated and secreted into the circulation. After interacting with FGFR1 in cardiomyocytes in the presence of *β*-klotho, FGF21 activates its downstream kinases and proteins including phosphatidylinositol 3-kinase (PI3K), protein kinase B (PKB/AKT), and Bcl2 antagonist of cell death (BAD), thereby reducing myocardial ischemia-induced apoptosis characterized by reduction of caspase-3 activity [59]. However, the adaptive response was not found in FGF21-deficient mice. Reversely, myocardial ischemic size was significantly smaller in FGF21 transgenic mice than that in wild type mice [59], suggesting that upregulated endogenous FGF21 derived from the liver and adipose tissue in response to myocardial injury induced cardiac protection mediated by activation of FGFR1/*β*-klotho-PI3K-Akt1-BAD signaling pathway. Although various growth factors and cytokines were upregulated during myocardial ischemia, the expression and secretion of cardiac FGF21 had no alteration, implying FGF21 induces cardiac protection against myocardial ischemia in an endocrine rather than an autocrine manner [59, 60]. To date, a question of whether administration of exogenous FGF21 can also induce cardiac protection during myocardial ischemia and if so whether the protection of exogenous FGF21 against myocardial ischemia can be direct to the heart or cardiomyocytes appears. This question was answered by Patel group [61]. They found that administration of exogenous FGF21 induced significant cardioprotection and restored cardiac function following global ischemia in Langendorff perfused rat hearts. Further study revealed that inhibition of AKT, extracellular signal-regulated kinase (ERK1/2), and AMPK impaired FGF21-induced antimyocardial ischemia effect in the hearts of obese Wistar rats, suggesting that the above kinases are involved in this cardioprotection of FGF21 [61]. Our previous* in vitro* study also confirmed that administration of exogenous FGF-21 attenuated ischemia-reperfusion induced damage in H9c2 cells characterized by inhibition of oxidative stress and apoptosis [62]. The mechanistic study revealed that FGF21-induced protection against ischemia-reperfusion injury in cardiac cells mainly depended on the activation of Akt-GSK-3*β*-caspase-3 signaling pathway by preventing oxidative stress and recovery of the energy supply [62].

## 4. FGF21 and Cardiac Hypertrophy 

Hypertrophic remodeling characterized by enlarged cardiomyocytes is an adaptive response of the heart to certain stresses. And it is also the leading cause of multiple cardiovascular problems including hypertension, myocardial ischemia, valvular disease, and cardiomyopathy [67–69]. Mature cardiomyocytes are considered to be terminally differentiated cells with no regenerative ability [70–72]. Under stresses, cardiac hypertrophy is characterized by cardiomyocytes enlargement, rather than cells division [73, 74], and this phenomenon is accompanied by the increase of extracellular matrix and fibroblasts inside the heart [75, 76].

Recently, cardiac hypertrophy was reported to induce FGF21 gene expression in the cardiomyocytes of mouse, and this was subjected to transcriptional regulation of the hepatic silent mating type information regulation 2 homolog 1/PPAR*α* pathway [64]. In turn, FGF21 knockout mice had greater heart weights and more severe cardiac dysfunction in response to isoproterenol infusion along with induction of hypertrophic inflammatory markers [64]. However, administration of recombinant FGF21 significantly prevented isoproterenol-induced cardiac hypertrophy damage in mice [64]. Mechanistic studies indicated that FGF21 prevented cardiac hypertrophy by activating mitogen-activated protein kinase (MAPK) signaling via activation of FGFR1c/*β*-klotho [64, 77]. Additionally, FGF21 prevented cardiac hypertrophy by promoting multiple antioxidant genes expressions (e.g., uncoupling proteins 2 and 3, also superoxide dismutase-2) and inhibiting the formation of reactive oxygen species in an autocrine manner [65].

## 5. FGF21 and Diabetic Cardiomyopathy

Diabetic patients develop the diabetic cardiomyopathy independent of coronary artery disease and hypertension [78, 79]. Diabetic cardiomyopathy is attributed to multiple pathogenic factors, including hyperglycemia, hyperlipidemia, and inflammation [80–82]. Cardiomyopathy is a late consequence of diabetes-induced early cardiac responses especially the myocardial apoptosis [83, 84]. Thus, treatments to reduce cardiac apoptosis may help control diabetic cardiomyopathy.

Recently, we reported that cardiac FGF21 mRNA expression was positively associated with the development of diabetes in the type 1 diabetic mice, suggesting that the increased cardiac FGF21 expression may be beneficial to the heart in this regard [30]. In the study we also observed cardiac apoptosis in early diabetic mice, which was remarkably prevented by administration of recombinant FGF21 [30]. Similar protection by FGF21 was observed in mice with cardiac lipotoxicity induced by fatty-acid [30]. Mechanistic studies indicated that FGF21-induced antiapoptotic effects* in vitro* and* in vivo* were mediated by ERK1/2-p38-MAPK-AMPK signaling pathway [30]. Thus, FGF21-induced cardioprotection in diabetic mice is mainly attributed to prevention of lipotoxicity by FGF21. Also, long-term treatment of FGF21 prevented diabetic-induced cardiac dysfunction and fibrosis mediated by the same signaling pathway as above [30]. Our work also revealed that FGF21 deletion-aggravated cardiac lipid accumulation is likely mediated by cardiac Nrf2-driven CD36 upregulation in type 1 diabetic mice, which contributes to increased cardiac oxidative stress and remodeling, and eventual development of diabetic cardiomyopathy [66].

## 6. Summary

**Figure 1:**
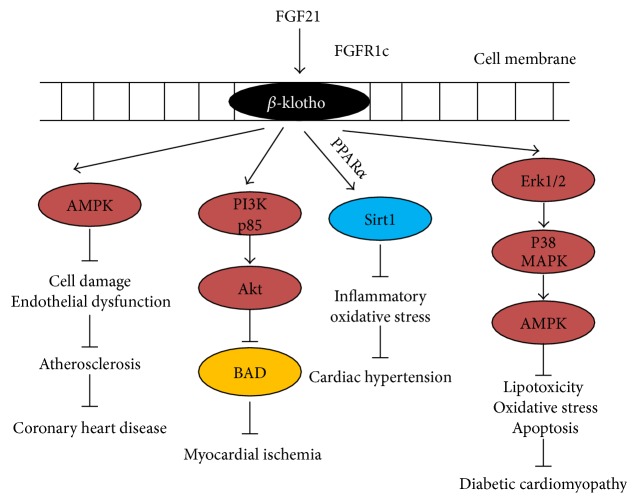
FGF21 induces preventive effect on CVDs through multiple signaling pathways. As a classical cytokine, FGF21 functions as a metabolic regulator by binding with its receptor FGFR1 or FGFR2 in the presence of *β*-klotho. Growing studies demonstrated that FGF21 also induced beneficial effects on CVDs probably due to inhibition of glucose or lipid metabolic disorders. For instance, FGF21 prevented atherosclerosis and the subsequent CHD by inhibition of lipogenesis which was also the possible mechanism of FGF21-induced preventive effect on CH. Additionally, FGF21 also prevented MI and DC by activation of Akt- and AMPK-mediated signaling pathway which were usually involved in maintaining glucose and lipid homeostasis.

CVD includes atherosclerosis, coronary heart disease, myocardial ischemia, cardiac hypertrophy, and diabetic cardiomyopathy which are all closely associated with severe lipid metabolic disorders [85–87]. FGF21, a metabolic regulator of carbohydrates and lipids, has been shown to improve insulin sensitivity and glucose uptake and suppress lipogenesis and lipid oxidation [15–18]. Clinical studies indicated that serum FGF21 changes were positively associated with the development of atherosclerosis, coronary heart disease, myocardial ischemia, cardiac hypertrophy, and diabetic cardiomyopathy, which implies that upregulated endogenous FGF21 may improve CVDs. Specifically, FGF21 prevented atherosclerosis and subsequent coronary heart disease was attributed to multiorgan cross talk among the liver, adipose tissue, and blood vessels and was characterized by suppression of lipid accumulation and increased lipid oxidation [63]. Similarly, FGF21 prevented stress-induced CH via enhancing lipid oxidation mediated by the ERK1/2-CREB-PGC-1*α* signaling pathway [64]. FGF21 also prevented myocardial ischemia and diabetic cardiomyopathy via Akt- or AMPK-mediated signaling pathways which regulate lipid and glucose metabolisms (Figure 1). Since serum FGF21 increases in several kinds of CVDs, serum FGF21 levels might be regarded as a potential biomarker not only for diagnosis of metabolic disorders but also for diagnosis of CVD in clinics. And supplementation of exogenous FGF21 might also induce beneficial effect in patients with CVD based on the conclusion of preclinical studies.

## Figures and Tables

**Table 1:** Summary of major pharmacological studies of FGF21 in heart disease.

Heart disease	Model	Methods	Outcomes	Ref.
Atherosclerosis	Apolipoprotein E^(−/−)^ mice	Recombinant murine FGF21 was given daily intraperitoneally for 16 weeks	Atherosclerotic lesion area collagen composition ↓ Total cholesterol ↓ Hypertriglyceridemia ↓ Circulating adiponectin ↑	[63]

Coronary heart disease		Mouse FGF21 full length protein was given for 24 or 48 hours	Cell apoptosis ↓ Oxidative stress ↓ NO production ↑ eNOS phosphorylation ↑	[55]

Myocardial ischemia	Coronary artery ligation (ischemia/reperfusion)	Recombinant mouse FGF21 was administered intravenously immediately after myocardial injury every 12 hrs for 3 days	Activity of caspase-3 ↓ Degree of myocardial infarction ↓ Left ventricular function ↑	[59]

Cardiac hypertrophy	Isoproterenol infusion-induced cardiac hypertrophy/LPS-induced cardiac hypertrophy	FGF21 was injected intraperitoneally for 7 days or given for 24 hours in neonatal cardiomyocytes	Cardiomyocyte size ↓ Δ heart weight/body weight ↓ Inflammation ↓ Cardiac oxidative stress ↓	[64, 65]

Diabetic cardiomyopathy	Multiple low-dose STZ-induced type 1 diabetes	Knockout FGF21 in type 1 diabetic mouse model	Oxidative stress ↑ Lipid accumulation ↑ Cardiac dysfunction and remodeling ↑	[66]
